# Exogenous Application of ABA and NAA Alleviates the Delayed Coloring Caused by Puffing Inhibitor in Citrus Fruit

**DOI:** 10.3390/cells10020308

**Published:** 2021-02-03

**Authors:** Gang Ma, Lancui Zhang, Rin Kudaka, Hayato Inaba, Takuma Furuya, Minami Kitamura, Yurika Kitaya, Risa Yamamoto, Masaki Yahata, Hikaru Matsumoto, Masaya Kato

**Affiliations:** 1 Department of Bioresource Sciences, Faculty of Agriculture, Shizuoka University, 836 Ohya, Suruga, Shizuoka 422-8529, Japan; ma.gang@shizuoka.ac.jp (G.M.); zhang.lan.cui@shizuoka.ac.jp (L.Z.); yahata.masaki@shizuoka.ac.jp (M.Y.); 2 Graduate School of Integrated Science and Technology, Shizuoka University, 836 Ohya, Suruga, Shizuoka 422-8529, Japan; twentyfour.08@icloud.com (R.K.); inaba6991@gmail.com (H.I.); furuya-takuma@zennoh.or.jp (T.F.); cherry37rei@yahoo.co.jp (M.K.); yurika_kitaya@hotmail.co.jp (Y.K.); Yamamoto.risa.15@shizuoka.ac.jp (R.Y.); 3 National Institute of Fruit Tree Science (NIFTS), National Agriculture and Bio-Oriented Research Organization (NARO), Shizuoka 424-0292, Japan; hikaruoo@affrc.go.jp

**Keywords:** carotenoid, chlorophyll, citrus fruit, peel puffing, plant hormone

## Abstract

Combined spraying of gibberellin (GA) and prohydrojasmon (PDJ) was an effective method to reduce peel puffing in Satsuma mandarins. However, in the GA-and-PDJ combined treatment, fruit color development was delayed during the ripening process. In the present study, to improve the coloration of the GA and PDJ-treated fruit, the effects of exogenous application of 1-naphthaleneacetic acid (NAA) and abscisic acid (ABA) on chlorophyll and carotenoid accumulation were investigated. The results showed that both ABA and NAA treatments accelerated the color changes from green to orange in the GA and PDJ-treated fruit during the ripening process. With the NAA and ABA treatments, chlorophylls contents were decreased rapidly, and the contents of β,β-xanthophylls were significantly enhanced in the GA and PDJ-treated fruit. In addition, gene expression results showed that the changes of the chlorophyll and carotenoid metabolisms in the NAA and ABA treatments were highly regulated at the transcriptional level. The results presented in this study suggested that the application of NAA and ABA could potentially be used for improving the coloration of the GA and PDJ-treated fruit.

## 1. Introduction

Peel puffing is a citrus physiological disorder in which albedo breaks down and peel severely separates from the pulp. Peel puffing is often observed in some loose-skin varieties, such as mandarins, and the overripe fruit when left on the tree [1]. In Japan, peel puffing occurs in late autumn when the peel continues growing after the pulp has completed development. Peel puffing significantly affects fruit quality and taste, which leads to the fruit becoming unappealing to consumers and reduces the fruit marketability. Moreover, puffy fruit cannot be stored for a long period, as it is easily damaged during harvesting, packing, and transporting, and is susceptible to postharvest decay [1,2,3,4]. In past decades, it has been suggested that combined spraying of gibberellin (GA) and prohydrojasmon (PDJ) was an effective method to reduce peel puffing in Satsuma mandarins [2,3,5]. However, in the GA-and-PDJ combined treatment, the fruit color development was delayed during the ripening process. The negative effect of the combined spraying of GA and PDJ on fruit coloring significantly limited its application in peel puffing.

**Figure 1:**
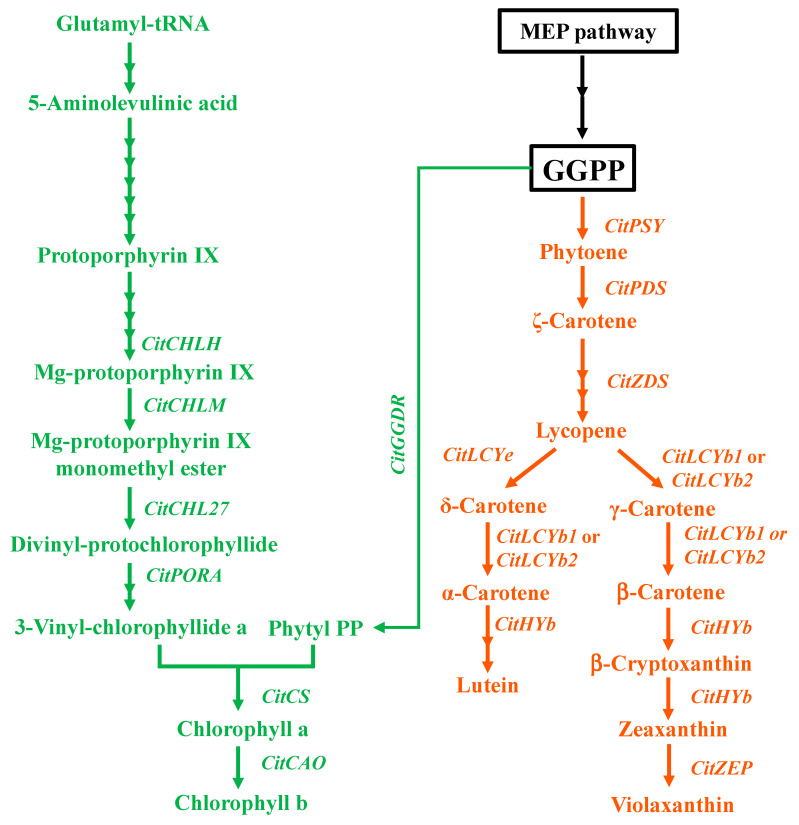
The biosynthetic pathways of chlorophyll and carotenoid from methylerthritol-4-phosphate (MEP) in plants. MEP pathway, methylerthritol-4-phosphate pathway; GGPP, geranylgeranyl diphosphate. The enzymes investigated in this study are: CHLH, magnesium chelatase; CHLM, magnesium-protoporphyrin IX methyltransferase; CHL27, Mg-Proto IX monomethyl ester cyclase; PORA, protochlorophyllide oxidoreductase A; CS, chlorophyll synthase; CAO, chlide a oxygenase; GGDR, geranylgeranyl reductase; PSY, phytoene synthase; PDS, phytoene desaturase; ZDS, ζ-carotene desaturase; LCYb, lycopene β-cyclase; LCYe, lycopene ε-cyclase; HYb, β-ring hydroxylase; ZEP, zeaxanthin epoxidase.

In citrus, the peel color changing from green to orange is a typical characteristic of fruit maturation [6]. In most citrus fruit, chlorophyll and carotenoid are two main groups of pigments that are responsible for the peel color. During the fruit ripening, the contents of chlorophylls, which are predominately accumulated in the immature fruit, decrease rapidly to a low level in the peel, whereas the contents of carotenoids, especially β,β-xanthophylls, increase gradually during the fruit ripening [7,8,9,10]. In plants, chlorophyll and carotenoid share a common biosynthetic pathway [11,12,13] (Figure 1). The structure of chlorophyll contains two parts: a substituted porphyrin ring and a phytol chain. The porphyrin ring is synthesized from glutamate in chloroplasts through the cooperative activity of more than 17 enzymes, and the phytol chain is synthesized from the reduction of geranylgeranyl diphosphate (GGPP) catalyzed by geranylgeranyl reductase (GGDR) (Figure 1). In addition, the GGPP is also a common precursor of carotenoid. The condensation of two molecules of GGPP by phytoene synthase (PSY) produces phytoene, which is the first and rate-limiting step for carotenoid biosynthesis (Figure 1). The key genes related to chlorophyll and carotenoid biosyntheses in citrus have been extensively investigated in the past decades (Figure 1). It was widely acknowledged that transcriptional regulation is critical for regulating the chlorophyll and carotenoid accumulation in citrus fruit during the ripening process [7,14,15,16,17,18,19].

In citrus fruit, color development in the peel is regulated by the environmental and nutritional conditions, as well as plant hormone treatments [10,16,17,19,20,21,22,23]. In previous studies, it was reported that GA treatment delayed the color break and harvest date in various citrus varieties. Preharvest treatment with GA before the onset of color break was found to reduce the loss of chlorophylls and prevent the accumulation of carotenoids in the peel [10,15,24,25]. As the GA treatment has a negative effect on color development in citrus fruit, the combined spraying of GA and PDJ on tree led to mature fruit with non-uniform greenish color in the peel, which significantly limited the application of GA and PDJ in the peel puffing. 1-Naphthaleneacetic acid (NAA) and abscisic acid (ABA) are two important plant hormones that regulate plant growth and development. Recent studies suggested that NAA and ABA have a potential role in accelerating fruit ripening and enhancing color development in citrus fruit [26,27,28,29,30,31]. In this study, to improve the coloration of GA and PDJ-treated fruit, we treated the fruit with NAA and ABA on tree after spraying GA and PDJ. Moreover, the effects of NAA and ABA on the chlorophyll and carotenoid contents as well as the expression of genes related to chlorophyll and carotenoid biosyntheses in the flavedos of the GA and PDJ-treated fruit were discussed. The results presented in this study will not only be helpful for understanding the roles of NAA and ABA in the color development of citrus fruit but also provide a novel strategy for improving the coloration of GA and PDJ-treated fruit.

## 2. Materials and Methods

### 2.1. Plant Materials and Treatments

**Figure 2:**
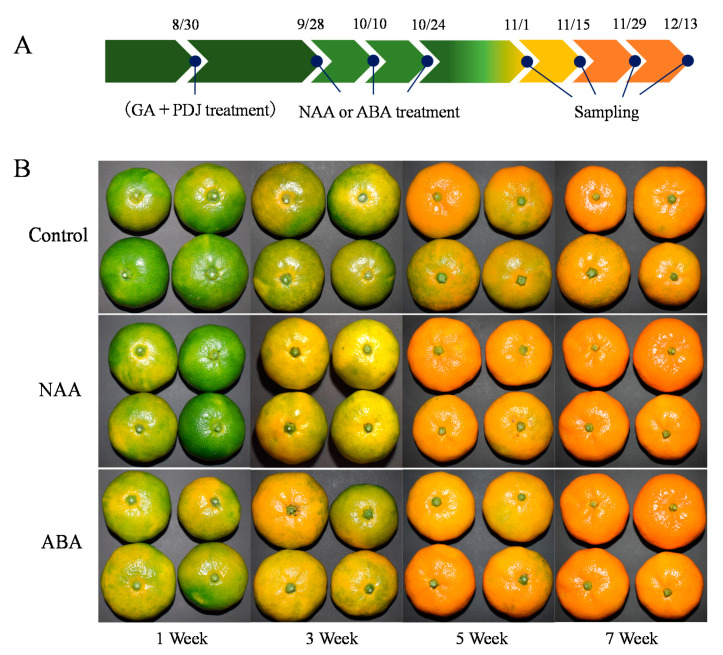
Effect of NAA and ABA on the appearance of gibberellin (GA) and prohydrojasmon (PDJ)-treated citrus fruit. (**A**) the treatment schedule; (**B**) the changes of the color in the peel. Fruit treated with GA and PDJ was used as control.

In this study, three 40-year-old trees of Satsuma mandarin “Aoshima unshiu” (*Citrus unshiu* Marcow.) in the Fujieda Farm of Shizuoka University (Shizuoka, Japan) were used as plant materials. On each tree, three branches in the medium part were separated by plastic sheets and labeled randomly as control, NAA, and ABA. Each branch bore approximately 60 fruit. In the present study, to prevent the occurrence of puffing, the fruit were sprayed on tree with a citrus puffing inhibitor mixture, which contained 5 mg L^−1^ of GA and 50 mg L^−1^ of PDJ, on 30 August 2018. After four weeks, the fruit were sprayed three times on tree with 500 µM of plant hormones NAA or ABA on 28 September, October 10th, and October 24th, respectively. To evaluate the effects of NAA and ABA on the fruit coloring, we sampled the fruit in the first week (1 November), third week (15 November), fifth week (29 November), and seventh week (13 December) after the NAA and ABA treatments, respectively (Figure 2A).

### 2.2. Color Measurement

On each sampling day, six fruit of each treatment were harvested randomly from the three trees. The peel color was determined with a colorimeter (NR-11, Nippon Denshoku, Japan) at 3 positions on the equatorial plane of each fruit, and the CIE 1976 (L*, a*, b*) color scale was used. The hue angle H° = arctangent (b*/a*) and citrus color index (CCI) = 1000 × a*/(L* × b*) were calculated according to methods described previously [32].

### 2.3. Extraction and Determination of Chlorophylls

Six fruit of each treatment were randomly harvested on each sampling day. The flavedos were separated from the sampled fruit, homogenized with a Shake Master (Bio Medical Science Inc. (BMS), Tokyo, Japan), and stored at −80 °C. The homogenized samples were used for chlorophyll, carotenoid, and gene expression analyses. Chlorophyll was extracted from the flavedos using 5 mL of *N*,*N*-dimethylformamide. After incubating overnight, the samples were centrifuged at 3000 rpm for 10 min. Then the absorbances of the supernatants were determined by spectrophotometer at 664 and 647 nm. The concentrations of chlorophyll a (Chl a), chlorophyll b (Chl b), and total chlorophyll were calculated according to a previously described method [33]. Three extracts were prepared for chlorophyll quantification.

### 2.4. Extraction and Determination of Carotenoids 

About 0.5 g of the frozen powder was used in carotenoid extraction. The extraction and determination of carotenoids in citrus flavedos were conducted using previously described methods [7]. Carotenoids were extracted from the flavedos using the extraction solution (hexane/acetone/ethanol, 2:1:1, *v*/*v*) containing 10% (*w*/*v*) magnesium carbonate basic. The organic solvents were evaporated by a rotary evaporator at a maximum temperature of 35 °C under a vacuum condition. After the organic solvents had been completely evaporated, the saponification was carried out by adding 8 mL 20% (*w*/*v*) methanolic potassium hydroxide (KOH) and 12 mL diethyl ether containing 0.1% (*w*/*v*) 2,6-di-*tert*-butyl-4-methylphenol. Then, the NaCl-saturated water was added to remove the water-soluble extracts. The carotenoids repartitioned into the diethyl ether phase were collected and evaporated to dryness. Subsequently, the residue was redissolved in a tert-butyl methyl ether (TBME)/methanol (1:1, *v*/*v*) solution and analyzed by HPLC. The HPLC was a reverse-phase HPLC system (Jasco, Tokyo, Japan) fitted with a YMC Carotenoid S-5 column (Waters, Milford, MA, USA). The contents of β-cryptoxanthin, lutein, all-*trans*-violaxanthin, and 9-*cis*-violaxanthin were analyzed by the standard curves and expressed as micrograms per gram of fresh weight [7]. The standards of lutein and all-*trans*-violaxanthin were obtained from DHI Water and Environment (Horsholm, Denmark). The standard of β-cryptoxanthin was obtained from Sokenkagaku (Tokyo, Japan). The standard of 9-*cis*-violaxanthin was prepared from the flavedo of Satsuma mandarin. The total carotenoid was calculated by summing all identified carotenoids. Three extracts were prepared for carotenoid quantification.

### 2.5. Total RNA Extraction and Real-Time Quantitative RT-PCR

About 1 g of the frozen powder was used in RNA extraction. Total RNA was extracted from the samples according to a previously described method [7]. The total RNA was purified using the RNeasy Mini Kit (Qiagen, Hilden, Germany), and on-column DNase digestion was performed. The quality and quantity of RNA were determined spectrophotometrically by measuring the OD_260/280_ and OD_260/230_. RNA integrity was assessed by visual inspection after electrophoresis on a formaldehyde agarose gel in the presence of ethidium bromide. The reactions of reverse transcription (RT) were performed with a random hexamer, 1 μg of purified RNA, and TaqMan Reverse Transcription Reagents at 37 °C (Applied Biosystems, Foster City, CA, USA). 

In this study, TaqMan MGB probes and sets of primers for chlorophyll biosynthetic genes (*CitGGDR*, *CitCHLH*, *CitCHLM*, *CitCHL27*, *CitPORA*, *CitCS*, and *CitCAO*) and carotenoid biosynthetic genes (*CitPSY*, *CitPDS*, *CitZDS*, *CitLCYb1*, *CitLCYb2*, *CitHYb*, *CitLCYe*, and *CitZEP)* were designed using the Primer Express software (Table S1). For the endogenous control, the TaqMan Ribosomal RNA Control Reagents VIC Probe (Applied Biosystems) was used. TaqMan real-time PCR was carried out by using TaqMan Universal PCR Master Mix (Applied Biosystems) and StepOnePlus^TM^ Real-Time PCR System (Applied Biosystems). Each reaction mixture contained 2 ng μL^−1^ of template cDNA, 250 nM of TaqMan MGB Probe, and 900 nM of primers. The thermal cycling conditions consisted of 95 °C for 10 min followed by 40 cycles of 95 °C for 15 s and 60 °C for 1 min. The expression levels of genes were calculated using the standard curve method. The standard curves were prepared for both the target and the endogenous reference (18S rRNA). For each experimental sample, the amount of target and endogenous reference was determined from the appropriate standard curve. Then, the target amount was divided by the endogenous reference amount to obtain a normalized target value. Three extracts were prepared for real-time quantitative RT-PCR analysis.

### 2.6. Statistical Analysis

All values are shown as the mean ± standard error (SE). The data were analyzed, and Tukey’s honest significant difference (HSD) test was used to analyze the differences in different treatments at *p* < 0.05 level. 

## 3. Results

### 3.1. Effects of NAA and ABA Spraying on the Color of the GA and PDJ-Treated Citrus Fruit

**Figure 3:**
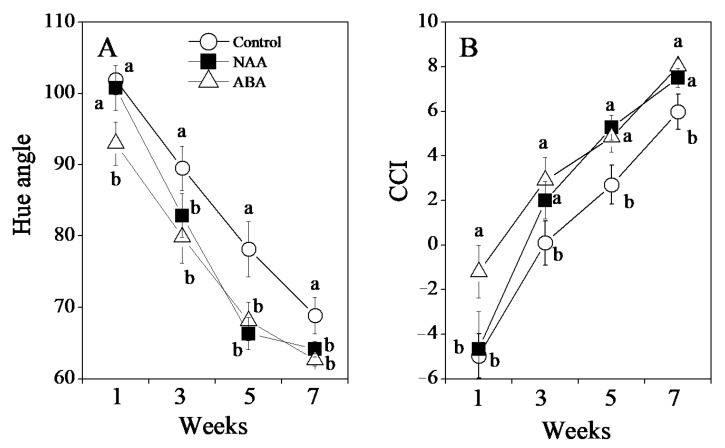
Effect of NAA and ABA on (**A**) hue angle and (**B**) citrus color index (CCI) in GA and PDJ-treated citrus fruit. The results shown are the mean ± SE (*n* = 6). Different letters (a and b) indicate significant differences at the 5% level by Tukey’s test.

In this study, the GA and PDJ-treated fruit were used as the control. As shown in Figure 2B, NAA and ABA treatments accelerated color development in the peel during the ripening process. In the third week, fruit treated with NAA and ABA had begun to turn orange, whereas the fruit of the control still retained light greenish color. In the seventh week, the fruit in the NAA and ABA treatment groups turned orange completely (Figure 2B). In the control, in contrast, the peel color was not uniform, with light green in the seventh week (Figure 2B). The changes of color in the peel were further analyzed by hue angle (H°) and CCI. With the color changing from green to orange, the hue angle (H°) decreased while the CCI increased in the control during the ripening process (Figure 3). In the NAA and ABA treatment groups, the changes in hue angle (H°) and CCI were significantly accelerated. During the ripening process, the hue angle (H°) in the NAA and ABA treatment groups decreased more rapidly than that in the control (Figure 3A). In the citrus industry, CCI is a standard parameter used to determine the color of citrus fruit; the negative value is for blue-green color and the positive value was for orange color. In the NAA and ABA treatment groups, the CCI was significantly increased during the ripening process and reached approximately 7.5 in the seventh week, which was higher than that of the control (5.9; Figure 3B). In addition, in the ABA treatment group, the color break occurred in the first week along with a significant decrease in hue angle (H°) and increase in CCI (Figure 2B and Figure 3). The appearance of the color break in the ABA treatment group was earlier than that in the NAA treatment group.

### 3.2. Effects of NAA and ABA Spraying on Chlorophyll Accumulation in the GA and PDJ-Treated Citrus Fruit

**Figure 4:**
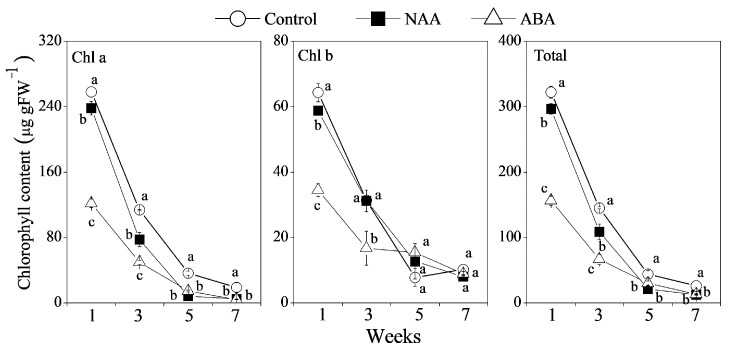
Effect of NAA and ABA on chlorophyll content in the flavedo of GA and PDJ-treated citrus fruit. Chl a, chlorophyll a; Chl b, chlorophyll b; Total, total chlorophyll. The results shown are the mean ± SE for triplicate samples. Different letters (a, b, and c) indicate significant differences at the 5% level by Tukey’s test.

During the ripening process, the contents of chlorophyll a, chlorophyll b, and total chlorophyll decreased gradually in the control. With the NAA and ABA treatments, the decreases in chlorophyll contents were accelerated during the ripening process (Figure 4). The total content of chlorophyll in the NAA and ABA treatment groups was much lower than that in the control during the ripening process. In addition, ABA treatment was more effective to induce chlorophyll reduction than NAA treatment. In the first week, the total chlorophyll content in the ABA treatment group was approximately half of that in the control (Figure 4).

**Figure 5:**
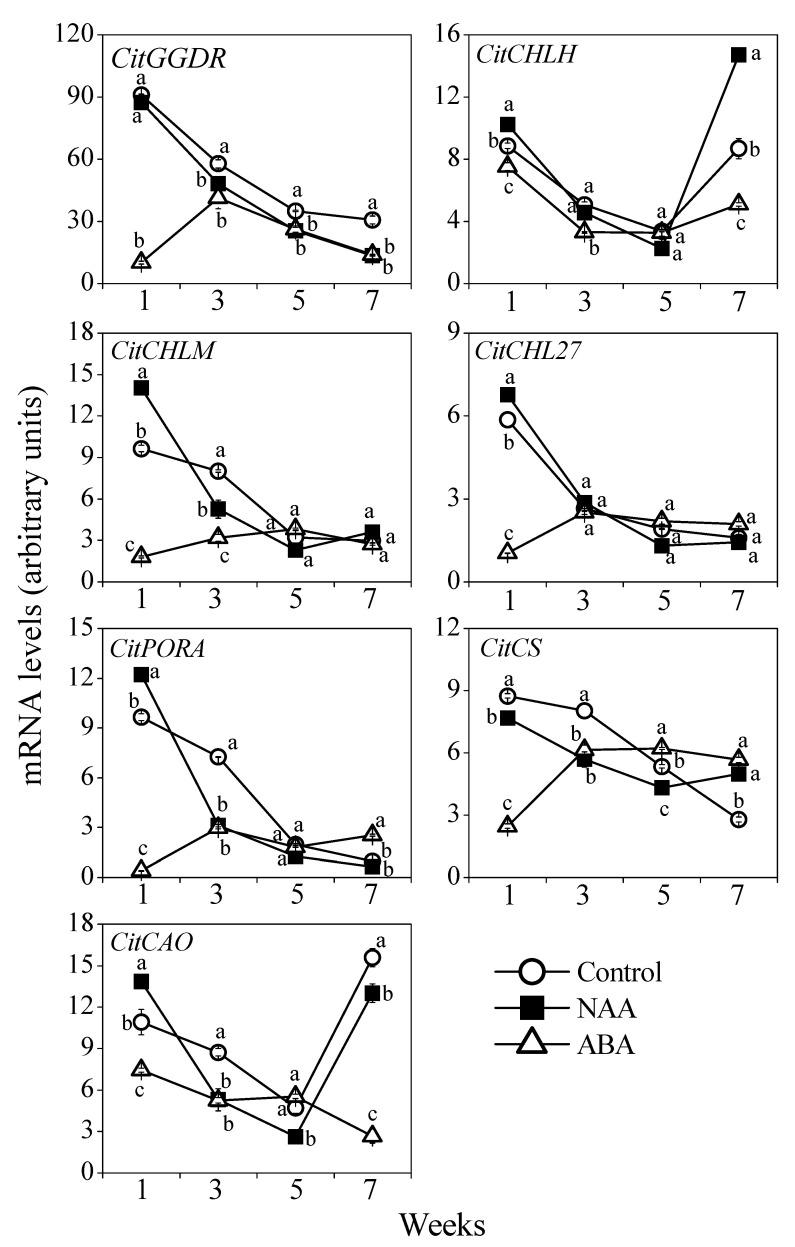
Effect of NAA and ABA on the expression of chlorophyll biosynthetic genes in the flavedo of GA and PDJ-treated citrus fruit. The mRNA levels were analyzed by TaqMan quantitative RT-PCR. RT-PCR amplification of 18S ribosomal RNA was used to normalize the expression of the genes under identical conditions. The results shown are the mean ± SE for triplicate samples. Different letters (a, b, and c) indicate significant differences at the 5% level by Tukey’s test.

In the present study, the expression of seven chlorophyll biosynthetic genes (*CitGGDR*, *CitCHLH*, *CitCHLM*, *CitCHL27*, *CitPORA*, *CitCS*, and *CitCAO*) was analyzed. The expression of chlorophyll biosynthetic genes decreased gradually in the control and NAA treatment group during the ripening process (Figure 5). In the NAA treatment group, the expression of *CitGGDR*, *CitCHLM*, *CitPORA*, *CitCS*, and *CitCAO* decreased more rapidly than the control. The expression levels of *CitGGDR*, *CitCHLM*, *CitPORA*, *CitCS*, and *CitCAO* in the NAA treatment group were significantly lower than the control in the third week (Figure 5). In the ABA treatment group, the expression of the chlorophyll biosynthetic genes kept constantly at a low level during the ripening process. In particular, the expression levels of *CitGGDR*, *CitCHLH*, *CitCHLM*, *CitCHL27*, *CitPORA*, *CitCS*, and *CitCAO* in the ABA treatment group were significantly lower than those of the control in the first week (Figure 5).

### 3.3. Effects of NAA and ABA Spraying on Carotenoid Accumulation in the GA and PDJ-Treated Citrus Fruit

**Figure 6:**
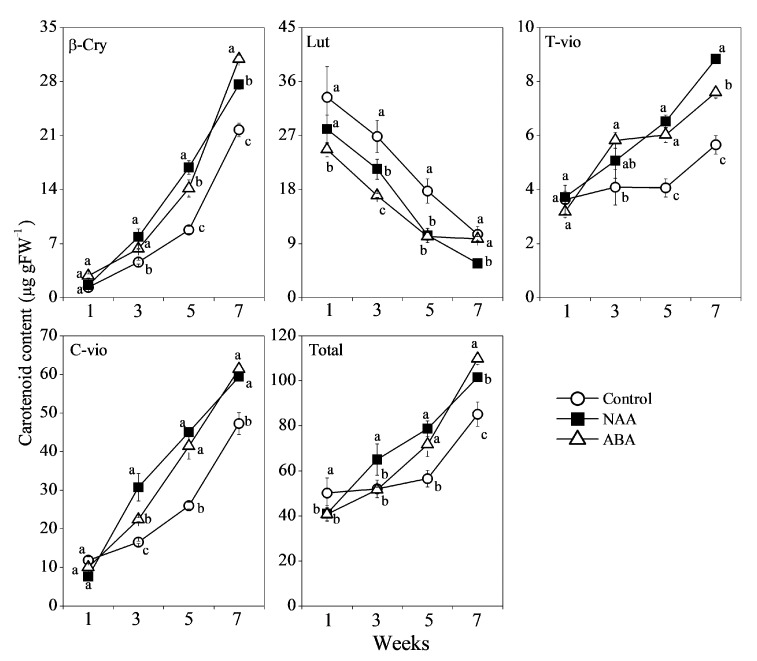
Effect of NAA and ABA on carotenoid content in the flavedo of GA and PDJ-treated citrus fruit. β-Cry, β-cryptoxanthin; Lut, lutein; T-vio, all-*trans*-violaxanthin; C-vio, 9-*cis*-violaxanthin; Total, total carotenoid. The value for total carotenoid was the sum of identified carotenoids. The results shown are the mean ± SE for triplicate samples. Different letters (a, b, and c) indicate significant differences at the 5% level by Tukey’s test.

In “Aoshima unshiu”, β-cryptoxanthin, lutein, all-*trans*-violaxanthin, and 9-*cis*-violaxanthin were the major carotenoids accumulated in the flavedo. During the ripening process, the content of lutein decreased rapidly in the control, while the contents of β-cryptoxanthin, all-*trans*-violaxanthin, and 9-*cis*-violaxanthin, increased gradually in the control (Figure 6). In the NAA and ABA treatment groups, the changes in the carotenoid composition were similar to that of the control. However, the content of lutein in the NAA and ABA treatment groups decreased more drastically than the control. The contents of β-cryptoxanthin, all-*trans*-violaxanthin, and 9-*cis*-violaxanthin were significantly increased by the NAA and ABA treatments during the ripening process, and as a result, the total carotenoid content in the flavedos treated with NAA and ABA was much higher than that of the control (Figure 6).

**Figure 7:**
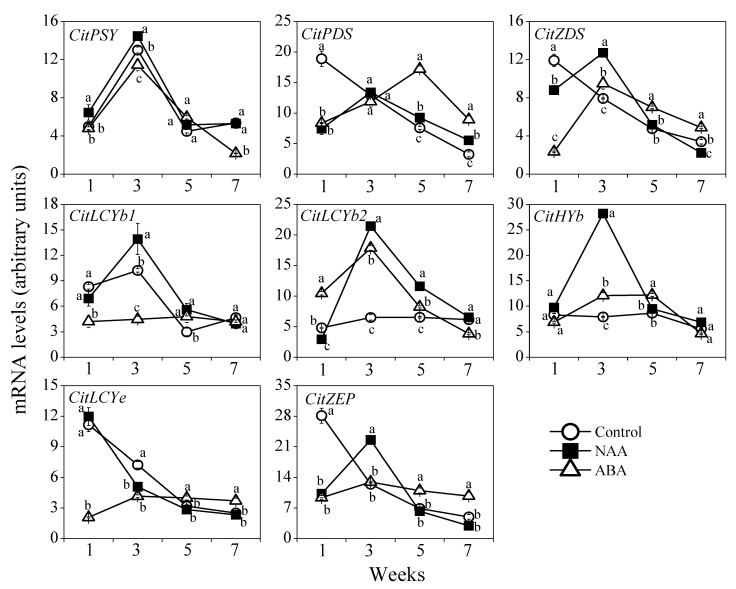
Effect of NAA and ABA on the expression of carotenoid biosynthetic genes in the flavedo of GA and PDJ-treated citrus fruit. The mRNA levels were analyzed by TaqMan quantitative RT-PCR. RT-PCR amplification of 18S ribosomal RNA was used to normalize the expression of the genes under identical conditions. The results shown are the mean ± SE for triplicate samples. Different letters (a, b, and c) indicate significant differences at the 5% level by Tukey’s test.

The gene expression results showed that the expression of *CitPSY* and *CitLCYb1* increased with a peak in the third week, while the expression of *CitPDS*, *CitZDS*, *CitLCYe*, and *CitZEP* decreased during the ripening process in the control. The expression of *CitLCYb2* and *CitHYb* in the control remained almost unchanged during the ripening process (Figure 7). In the NAA treatment group, the expression of *CitPSY*, *CitPDS*, *CitZDS*, *CitLCYb1*, *CitLCYb2*, *CitHYb*, and *CitZEP* increased with a peak in the third week, while the expression of *CitLCYe* decreased rapidly during the ripening process. The expression levels of *CitPSY*, *CitZDS*, *CitLCYb1*, *CitLCYb2*, *CitHYb*, and *CitZEP* in the NAA treatment group were higher than that of the control (Figure 7). In the ABA treatment group, the expression of *CitPSY*, *CitZDS*, *CitLCYb2*, and *CitHYb* increased with a peak in the third week, and the expression of *CitPDS* increased with a peak in the fifth week. The expression of *CitLCYb1*, *CitLCYe*, and *CitZEP* in the ABA treatment group did not significantly change during the ripening process. Compared with the control, the expression levels of *CitPDS*, *CitZDS*, *CitLCYb2*, *CitHYb*, and *CitZEP* were higher in the ABA treatment group (Figure 7).

## 4. Discussion

Peel puffing is a serious physiological disorder in citrus that affects fruit quality and storage [1,2,3,4]. The occurrence of peel puffing is associated with water exchange regulation through the peel and nitrogen fertilization, and it is promoted by high temperature and humidity [2,34]. Along with global warming, peel puffing has become a big problem in citrus production in Japan [35]. GA and PDJ are two plant hormones that regulate key processes of plant growth and development. It has been reported that the combined spraying of GA and PDJ is an effective method to reduce peel puffing in citrus [2,3,5]. The combined spraying of GA and PDJ significantly decreased the puffing rate in Satsuma mandarins (Figure S1). However, the combined spraying of GA and PDJ delayed peel coloring during the ripening process, which led to mature fruit with non-uniform greenish color (Figure S1). The negative effects of combined spraying of GA and PDJ on the peel coloring limited its application in peel puffing. In the present study, to improve the peel coloration of GA and PDJ-treated fruit, the roles of NAA and ABA treatments in the peel coloring were investigated. The results showed that NAA and ABA treatments accelerated the color development in the peel of GA and PDJ-treated fruit during the ripening process (Figure 2). In the NAA and ABA treatment groups, CCI increased at a higher rate during the ripening process and reached a higher value than the control in the seventh week (Figure 3B). Moreover, NAA and ABA treatments did not diminish the protective effects of GA and PDJ treatment on puffing. The fruit did not develop puffing after NAA and ABA treatments. In addition, the appearance of the color break was obviously accelerated by the ABA treatment, which occurred in the first week and was earlier than that in the NAA treatment group and control (Figure 2). 

In citrus fruit, color development during the ripening process is a result of the rapid decrease of chlorophylls and accumulation of carotenoids. In the previous studies, it was found that the peel coloration could be improved by ethylene, red LED light, and low temperature through the regulation of chlorophylls and carotenoids accumulation [10,16,17,20,21,23,32]. In contrast, GA treatment was reported to inhibit the color development in citrus fruit. In the GA treatment, the color break was delayed, and the chlorophyll content and the green color were maintained longer during the fruit ripening process [10,15,24]. In the present study, the results showed that NAA and ABA treatments accelerated the loss of chlorophyll in the GA and PDJ-treated fruit during the ripening process, which contributed to improving the coloration of the GA and PDJ-treated fruit (Figure 4). In particular, the ABA treatment was found to be quite effective to induce chlorophyll decrease in the GA and PDJ-treated fruit. The chlorophyll content in the ABA treatment group decreased rapidly to approximately half of the control in the first week (Figure 4). The rapid decrease of chlorophyll in the first week was consistent with the earlier appearance of the color break in the ABA treatment group (Figure 2).

In plants, the genes related to chlorophyll biosynthesis have been extensively investigated in the past decades, and it was suggested that transcriptional regulation was a key mechanism that controlled the metabolic flux of chlorophyll [11,12,13,15,19]. In the present study, the results showed that the expression of chlorophyll biosynthetic genes (*CitGGDR*, *CitCHLH*, *CitCHLM*, *CitCHL27*, *CitPORA*, *CitCS*, and *CitCAO*) decreased gradually in the control during the ripening process (Figure 5). In the NAA treatment group, the expression of *CitGGDR*, *CitCHLM*, *CitPORA*, *CitCS*, and *CitCAO* decreased more dramatically, and their expression levels were lower than those of the control in the third week (Figure 5). The low expression levels of chlorophyll biosynthetic genes might lead to the decrease of chlorophyll in the NAA treatment group. In addition, in the present study, we found that the ABA treatment was more effective to repress the expression of the chlorophyll biosynthetic genes than the NAA treatment. In the ABA treatment group, the expression of *CitGGDR*, *CitCHLH*, *CitCHLM*, *CitCHL27*, *CitPORA*, *CitCS*, and *CitCAO* was significantly downregulated, and their expression levels were much lower than those of the control and NAA treatment group in the first week. The significant down-regulation of the chlorophyll biosynthetic genes was well consistent with the rapid decrease of chlorophylls in the first week after the ABA treatment. To date, the research on the chlorophyll biosynthesis during degreening in citrus fruit is still limited. In the present study, the results showed that the expression of chlorophyll biosynthetic genes decreased rapidly in the flavedo during the fruit degreening. Moreover, the expression of the chlorophyll biosynthetic genes was significantly downregulated by the NAA and ABA treatments, which was in agreement with the reduction in chlorophyll content (Figure 4 and Figure 5). These results suggested that the inhibition of chlorophyll biosynthesis might have been an important mechanism that triggered the loss of chlorophyll in citrus fruit.

In the past decades, the roles of plant hormones in carotenoid biosynthesis have been extensively investigated in citrus fruit [10,16,17,32,36]. It was reported that GA had a negative effect on carotenoid biosynthesis in citrus fruit during the ripening process. In the GA treatment, the carotenoid biosynthesis from ε,β-branch to β,β-branch was delayed, which led to maintaining a higher content of lutein and prevented the accumulation of β,β-xanthophylls [10,15,16]. In contrast to GA, ABA, which is an end product of carotenoid biosynthesis, was effective to induce carotenoid accumulation in citrus fruit. During the ripening process, the carotenoid biosynthesis was accompanied by an increase in ABA content in the peel of citrus fruit. The endogenous accumulation of ABA as well as the exogenous ABA treatment was reported to play an important role in inducing carotenoid accumulation in citrus fruit [26,27,28,30]. Different from the research on GA and ABA, the research on the effects of NAA on carotenoid biosynthesis in citrus fruit has not been reported. In the present study, the results showed that both NAA and ABA treatments were effective to improve the carotenoid accumulation in the GA and PDJ-treated fruit (Figure 6). In the NAA and ABA treatment groups, the decrease in the content of lutein was accelerated, and the accumulation of β,β-xanthophylls (β-cryptoxanthin, all-*trans*-violaxanthin, and 9-*cis*-violaxanthin) was significantly induced in the flavedos during the ripening process. In addition, gene expression results showed that the expression of *CitLCYb2* and *CitHYb*, which were the two key genes regulating β,β-xanthophylls biosynthesis, remained constant at a low level in the control during the ripening process. Whereas, the expression of *CitLCYb2* and *CitHYb* was significantly upregulated by the NAA and ABA treatments (Figure 7). The higher expression levels of *CitLCYb2* and *CitHYb* in the NAA and ABA treatment groups led to the enhancement of β,β-xanthophylls contents [37,38,39]. In citrus fruit, β,β-xanthophylls were the main pigments responsible for the bright orange color of the peel in the mature fruit [9,40]. In the present study, the higher contents of β,β-xanthophylls in the NAA and ABA treatment groups contributed to improving the peel coloration in the GA and PDJ-treated fruit. 

## 5. Conclusions

In the present study, the effects of NAA and ABA treatments on the coloration of the GA and PDJ-treated fruit were investigated. The results showed that NAA and ABA treatments effectively improved the color development in the GA and PDJ-treated fruit. During the ripening process, the decrease in chlorophyll was accelerated, and the accumulation of β,β-xanthophylls was significantly enhanced by the NAA and ABA treatments in the GA and PDJ-treated fruit. The results presented in this study suggest that the spraying of NAA and ABA has great potential in improving the poor coloration of GA and PDJ-treated fruit, which might provide novel insights into the application of GA and PDJ in puffy fruit.

## Data Availability

Data is contained within the article or supplementary material.

## Supplementary Materials

The following are available online at https://www.mdpi.com/2073-4409/10/2/308/s1, Figure S1: Effect of combined spraying of GA and PDJ on the appearance of citrus fruit (November), Table S1: Primer sequences and TaqMan MGB probes used for the TaqMan RT-PCR of the genes related to chlorophyll and carotenoid biosynthesis.

## References

[1] Martinelli F., Ibanez A.M., Reagan R.L., Davino S., Dandekar A.M. (2015). Stress responses in citrus peel: Comparative analysis of host responses to Huanglongbing disease and puffing disorder. Sci. Hortic..

[2] Kawase K., Suzuki K., Hirose K. (1981). Use of Growth Regulators to Control Rind Puffing in Satsuma Mandarin Fruit.

[3] Garcialuis A., Agusti M., Almela V., Romero E., Guardiola J.L. (1985). Effect of gibberellic-acid on ripening and peel puffing in satsuma mandarin. Sci. Hortic..

[4] Shiraishi M., Mohammad P., Makita Y., Fujibuchi M., Manabe T. (1999). Effects of calcium compounds on fruit puffing and the ultrastructural characteristics of the subepidermal cell walls of puffy and calcium-induced non-puffy Satsuma Mandarin fruits. J. Jap. Soc. Hortic. Sci..

[5] Sato K., Ikoma Y., Matsumoto H., Nakajima N. (2015). Effects of spray concentration and spray times of gibberellin and prohydrojasmon on peel puffing and peel color in Satsuma mandarin fruit. Hort. Res..

[6] Iglesias D.J., Cercos M., Colmenero-Flores J.M., Naranjo M.A., Rios G., Carrera E., Ruiz-Rivero O., Lliso I., Morillon R., Tadeo F.R. (2007). Physiology of citrus fruiting. Braz. J. Plant Physiol..

[7] Kato M., Ikoma Y., Matsumoto H., Sugiura M., Hyodo H., Yano M. (2004). Accumulation of carotenoids and expression of carotenoid biosynthetic genes during maturation in citrus fruit. Plant Physiol..

[8] Tadeo F.R., Cercos M., Colmenero-Flores J.M., Iglesias D.J., Naranjo M.A., Rios G., Carrera E., Ruiz-Rivero O., Lliso I., Morillon R. (2008). Molecular physiology of development and quality of citrus. Adv. Bot. Res..

[9] Ríos G., Naranjo M.A., Rodrigo M.J., Alós E., Zacarías L., Cercós M., Talón M. (2010). Identification of a GCC transcription factor responding to fruit colour change events in citrus through the transcriptomic analyses of two mutants. BMC Plant Biol..

[10] Rodrigo M.J., Alquézar B., Alós E., Medina V., Carmona L., Bruno M., Al-Babili S., Zacarías L. (2013). A novel carotenoid cleavage activity involved in the biosynthesis of citrus fruit-specific apocarotenoid pigments. J. Exp. Bot..

[11] Bouvier F., Rahier A., Camara B. (2005). Biogenesis, molecular regulation and function of plant isoprenoids. Prog. Lipid Res..

[12] Pulido P., Perello C., Rodríguez-Concepción M. (2012). New insights into plant isoprenoid metabolism. Mol. Plant.

[13] Kobayashi K., Masuda T. (2016). Transcriptional regulation of tetrapyrrole biosynthesis in Arabidopsis thaliana. Front. Plant Sci..

[14] Rodrigo M.J., Marcos J.F., Zacarías L. (2004). Biochemical and molecular analysis of carotenoid biosynthesis in flavedo of orange (*Citrus sinensis* L.) during fruit development and maturation. J. Agric. Food. Chem..

[15] Alós E., Cercós M., Rodrigo M.J., Zacarías L., Talón M. (2006). Regulation of color break in citrus fruits. Changes in pigment profiling and gene expression induced by gibberellins and nitrate, two ripening retardants. J. Agric. Food Chem..

[16] Rodrigo M.J., Zacarías L. (2007). Effect of postharvest ethylene treatment on carotenoid accumulation and the expression of carotenoid biosynthetic genes in the flavedo of orange (*Citrus sinensis* L. Osbeck) fruit. Postharvest Biol. Technol..

[17] Ma G., Zhang L.C., Matsuta A., Matsutani K., Yamawaki K., Yahata M., Wahyudi A., Motohashi R., Kato M. (2013). Enzymatic formation of β-citraurin from β-cryptoxanthin and zeaxanthin by carotenoid cleavage dioxygenase4 in the flavedo of citrus fruit. Plant Physiol..

[18] Wei X., Chen C., Yu Q., Gady A., Yu Y., Liang G., Gmitter F.G. (2014). Comparison of carotenoid accumulation and biosynthetic gene expression between Valencia and Rohde Red Valencia sweet oranges. Plant Sci..

[19] Ma G., Zhang L.C., Kitaya Y., Seoka M., Kudaka R., Yahata M., Yamawaki K., Shimada T., Fujii H., Endo T. (2021). Blue LED light induces regreening in the flavedo of Valencia orange in vitro. Food Chem..

[20] Matsumoto H., Ikoma Y., Kato M., Nakajima N., Hasegawa Y. (2009). Effect of postharvest temperature and ethylene on carotenoid accumulation in the flavedo and juice sacs of Satsuma Mandarin (*Citrus unshiu* Marc.) fruit. J. Agric. Food Chem..

[21] Ma G., Zhang L.C., Kato M., Yamawaki K., Kiriiwa Y., Yahata M., Matsumoto H. (2012). Effect of blue and red LED light irradiation on β-cryptoxanthin accumulation in the flavedo of citrus fruits. J. Agric. Food Chem..

[22] Lado J., Alós E., Manzi M., Cronje P.J.R., Gómez-Cadenas A., Rodrigo M.J., Zacarías L. (2019). Light regulation of carotenoid biosynthesis in the peel of mandarin and sweet orange fruits. Front. Plant Sci..

[23] Mitalo O.W., Otsuki T., Okada R., Obitsu S., Masuda K., Hojo Y., Matsuura T., Mori I.C., Abe D., Asiche W.O. (2020). Low temperature modulates natural peel degreening in lemon fruit independently of endogenous ethylene. J. Exp. Bot..

[24] García-Luís A., Herrero-Villén A., Guardiola J.L. (1992). Effects of applications of gibberellic acid on late growth, maturation and pigmentation of the Clementine mandarin. Sci. Hortic..

[25] Fujii H., Shimada T., Sugiyama A., Endo T., Nishikawa F., Nakano M., Ikoma Y., Shimizu T., Omura M. (2008). Profiling gibberellin (GA3)-responsive genes in mature mandarin fruit using a citrus 22 K oligoarray. Sci. Hortic..

[26] Valero D., Martínez-Romero D., Serrano M., Riquelme F. (1998). Influence of postharvest treatment with putrescine and calcium on endogenous polyamines, firmness, and abscisic acid in lemon (*Citrus lemon* L. Burm cv. Verna). J. Agric. Food Chem..

[27] Rodrigo M.J., Marcos J.F., Alférez F., Mallent M.D., Zacarías L. (2003). Characterization of Pinalate, a novel Citrus sinensis mutant with a fruit specific alteration that results in yellow pigmentation and decreased ABA content. J. Exp. Bot..

[28] Wang X., Yin W., Wu J., Chai L., Yi H. (2016). Effects of exogenous abscisic acid on the expression of citrus fruit ripening-related genes and fruit ripening. Sci. Hortic..

[29] Bermejo A., Granero B., Mesejo C., Reig C., Tejedo V., Agustí M., Primo-Millo E., Iglesias D.J. (2018). Auxin and gibberellin interact in citrus fruit set. J. Plant Growth Regul..

[30] Rehmana M., Singha Z., Khurshid T. (2018). Pre-harvest spray application of abscisic acid (S-ABA) regulates fruit colour development and quality in early maturing M7 Navel orange. Sci. Hortic..

[31] Pérez-Llorca M., Muñoz P., Müller M., Munné-Bosch S. (2019). Biosynthesis, metabolism and function of auxin, salicylic acid and melatonin in climacteric and non-climacteric fruits. Front. Plant Sci..

[32] Ma G., Zhang L., Kato M., Yamawaki K., Kiriiwa Y., Yahata M., Ikoma Y., Matsumoto H. (2015). Effect of the combination of ethylene and red LED light irradiation on carotenoid accumulation and carotenogenic gene expression in the flavedo of citrus fruit. Postharvest Biol. Technol..

[33] Moran R. (1982). Formulae for determination of chlorophyllous pigments extracted with *N*,*N*-dimethylformamide. Plant Physiol..

[34] Sato K., Ikoma Y. (2020). Effects of elevated temperatures during the flowering to physiological fruit drop stage and at the fruit maturation stage on fruit quality of the satsuma mandarin. J. Agric. Meteorol..

[35] Sugiura T., Kuroda H., Sugiura H. (2007). Influence of the current state of global warming on fruit tree growth in Japan. Hort. Res..

[36] Zhang L.C., Ma G., Kato M., Yamawaki K., Takagi T., Kiriiwa Y., Ikoma Y., Matsumoto H., Yoshioka T., Nesumi H. (2012). Regulation of carotenoid accumulation and the expression of carotenoid metabolic genes in citrus juice sacs in vitro. J. Exp. Bot..

[37] Alquézar B., Zacarías L., Rodrigo M.J. (2009). Molecular and functional characterization of a novel chromoplast-specific lycopene β-cyclase from Citrus and its relation to lycopene accumulation. J. Exp. Bot..

[38] Zhang L., Ma G., Shirai Y., Kato M., Yamawaki K., Ikoma Y., Matsumoto H. (2012). Expression and functional analysis of two lycopene β-cyclases from citrus fruits. Planta.

[39] Ma G., Zhang L.C., Yungyuen W., Tsukamoto I., Iijima N., Oikawa M., Yamawaki K., Yahata M., Kato M. (2016). Expression and functional analysis of citrus carotene hydroxylases: Unraveling the xanthophyll biosynthesis in citrus fruits. BMC Plant Biol..

[40] Oberholster R., Cowan A.K., Molnar P., Toth G. (2001). Biochemical basis of color as an aesthetic quality in *Citrus sinensis*. J. Agric. Food Chem..

