# Supplementary material for: Exogenous Application of ABA and NAA Alleviates the Delayed Coloring Caused by Puffing Inhibitor in Citrus Fruit
Source: Cells. 2021 Feb 3;10(2):308. doi: 10.3390/cells10020308 (PMC7913354; doi:10.3390/cells10020308)
Supplement: Supplementary file 1 [file cells-10-00308-s001.pdf]

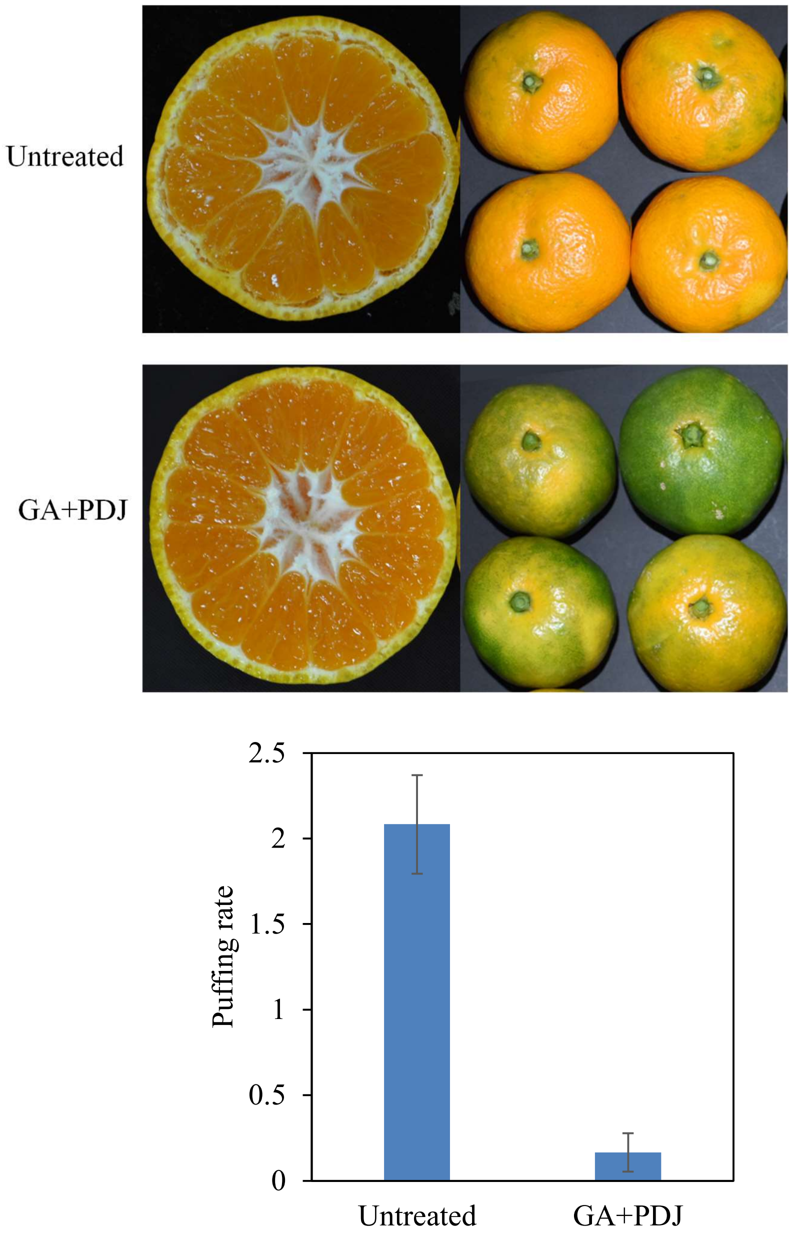

Figure S1. Effect of combined spraying of GA and PDJ on the appearance of citrus fruit (November). A, the puffing was inhibited by combined spraying of GA and PDJ, but the coloring of fruit was delayed. B, the puffing rate of fruit in the ripening stage (November). The level of peel puffing was assessed by finger touch and rated as 0, 1, 2, or 3, indicating a range from firm (0) to marked puffing (3) using the method described previously [34]. The results shown are the mean ± SE (n=12).

Table S1. Primer sequences and TaqMan MGB probes used for the TaqMan real-time RT-PCR of the genes related to chlorophyll and carotenoid biosynthesis.

Genes	Forward Primer	Reverse Primer	TaqMan MGB probe
CitGGDR	TGATGTGTCGCCGGACTTT	CCAACTGCCACATGATCACACT	ATGGGTGGGTGTTCC
CitCHLH	CCCACCACAGCAGCAATG	TGCCTCTCAATCAACCTATCCA	AGAGTGCAAAAGTTG
CitCHLM	AGTTGTTTCCTGGGCCTTCA	CAGCTTCGGCGTGGAGAT	AGGCAACGAGGGCT
CitCHL27	GAAAACCAACCCAGTGGTAGCT	GGCATGCCTGGCTTCATC	AGATTTTCTCTCTGATGTCC
CitPORA	TTTAGATCTGGCTTCCCTTGACA	GCCTGATCGCCTGAAGGTATC	TGTTCGCCAATTTG
CitCS	TACTCTGCTCCACCTTTGAAGCT	CTCGCTCCCAGAGCAAAATT	AGCAAAATGGATGGATTGG
CitCAO	TCGCATCCAATGCCCATAT	TTTCTCGCATTTCCCATCTGT	ATGGGTGGGAATACT
CitPSY	CGTTGATGGGCCTAATGCTT	ACCTGGACTCCCACCTGTCTAA	ACACATAACTCCAACAGC
CitPDS	TGGCAACCCCCCAGAGA	CACCCAGTGACTGAATGTGTT	ACTTTGCTTGCCTATTGT
CitZDS	AAAGGCACTTGTTGATCCTGATG	ACCAATCAGAGAAGCTTATACTATCCA	CCTTGAAGGACATACGAGAT
CitLCYb1	TGGTACCGCTGGGATGGT	CAATAGGAGCCGCAGCTAAAGT	CACCCTTCAACTGGCT
CitLCYb2	CCTTGGCTCAACCAGGATGA	ACCCATTCCACACTTTCTGATGA	CAGAGGCAGGCCAC
CitHYb	GCGGCTCACCAGCTTCAC	CCGAGAAAGAGCCCATATGG	ACTCGGATAAATTCC
CitLCYe	AAGGTGTGTCGAGTCAGGTGTTT	CCACTGGTAGATTCCGTAATGCT	ATATCTTAGCTCAAAAGTGG
CitZEP	CTAAAGAGCTATGAGAGAGCTAGGAGACT	CACTGCGGCCGATCTTG	CGAGTGGCTGTTATC
